# Identification of bottlenecks in the accumulation of cyclic fatty acids in camelina seed oil

**DOI:** 10.1111/pbi.12839

**Published:** 2018-01-18

**Authors:** Xiao‐Hong Yu, Rebecca E. Cahoon, Patrick J. Horn, Hai Shi, Richa R. Prakash, Yuanheng Cai, Maegan Hearney, Kent D. Chapman, Edgar B. Cahoon, Jorg Schwender, John Shanklin

**Affiliations:** ^1^ Department of Biochemistry and Cell Biology Stony Brook University Stony Brook NY USA; ^2^ Center for Plant Science Innovation Department of Biochemistry University of Nebraska‐Lincoln Lincoln NE USA; ^3^ Department of Biological Sciences BioDiscovery Institute University of North Texas Denton TX USA; ^4^ Biology Department Brookhaven National Laboratory Upton NY USA; ^5^ Present address: DOE‐Plant Research Laboratory Michigan State University East Lansing MI USA; ^6^ Present address: Department of Natural Sciences Suffolk County Community College Brentwood NY USA

**Keywords:** cyclopropane fatty acid, *Camelina sativa*, unusual fatty acid, lipid metabolism, triacylglycerol, lipid synthesis

## Abstract

Modified fatty acids (mFA) have diverse uses; for example, cyclopropane fatty acids (CPA) are feedstocks for producing coatings, lubricants, plastics and cosmetics. The expression of mFA‐producing enzymes in crop and model plants generally results in lower levels of mFA accumulation than in their natural‐occurring source plants. Thus, to further our understanding of metabolic bottlenecks that limit mFA accumulation, we generated transgenic *Camelina sativa* lines co‐expressing *Escherichia coli* cyclopropane synthase (EcCPS) and *Sterculia foetida* lysophosphatidic acid acyltransferase (SfLPAT). In contrast to transgenic CPA‐accumulating Arabidopsis, CPA accumulation in camelina caused only minor changes in seed weight, germination rate, oil accumulation and seedling development. CPA accumulated to much higher levels in membrane than storage lipids, comprising more than 60% of total fatty acid in both phosphatidylcholine (PC) and phosphatidylethanolamine (PE) versus 26% in diacylglycerol (DAG) and 12% in triacylglycerol (TAG) indicating bottlenecks in the transfer of CPA from PC to DAG and from DAG to TAG. Upon co‐expression of SfLPAT with EcCPS, di‐CPA‐PC increased by ~50% relative to lines expressing EcCPS alone with the di‐CPA‐PC primarily observed in the embryonic axis and mono‐CPA‐PC primarily in cotyledon tissue. EcCPS‐SfLPAT lines revealed a redistribution of CPA from the *sn*‐1 to *sn*‐2 positions within PC and PE that was associated with a doubling of CPA accumulation in both DAG and TAG. The identification of metabolic bottlenecks in acyl transfer between site of synthesis (phospholipids) and deposition in storage oils (TAGs) lays the foundation for the optimizing CPA accumulation through directed engineering of oil synthesis in target crops.

## Introduction

Modified fatty acids (mFAs) such as hydroxy, epoxy and conjugated fatty acids occur naturally in a limited number of plant species (herein referred to as source plants) which are generally unsuitable for mass agronomic production of the mFA (Haslam *et al*., 2016; Horn and Benning, 2016). The large potential market for mFAs has led to considerable interest in producing them in transgenic crops. However, compared to high levels found in source plants (e.g. castor bean oil contains ~90% of the hydroxy FA (ricinoleic acid)), crops transformed with genes encoding mFA‐synthesizing enzymes tend to accumulate relatively low levels (generally <20%) of the mFA (Bates, 2016; Haslam *et al*., 2016; Horn and Benning, 2016). Identifying bottlenecks in mFA accumulation in crop as well as model plants is therefore an essential step to designing new engineering strategies that will optimize their accumulation in future crops.

Plants and bacteria can accumulate cyclopropane fatty acids which contain a 3‐membered carbocyclic ring. The biosynthetic pathway of cyclopropane fatty acids (CPA‐FAs) was first defined in *E. coli* (Hildebrand and Law, 1964). The carbocyclic group is introduced by the action of the cyclopropane fatty acid synthase (CPS) enzyme which adds a methylene group from S‐adenosylmethionine, across a carbon–carbon double bond within a monounsaturated fatty acid esterified to phosphatidylethanolamine (PE) (Grogan and Cronan, 1997). A homolog from higher plants was identified in the seeds of *Sterculia foetida* which recognizes oleate esterified to phosphatidylcholine (PC) (Bao *et al*., 2002, 2003). CPA can be desaturated to form cyclopropene fatty acids (CPE‐FAs) which have potential applications as feedstocks for the production of lubricants, plastics, paints, dyes and coatings (Carlsson *et al*., 2011). Expression of plant CPS genes in tobacco led to the accumulation of only 1%–3% of 9,10‐methyleneoctadecanoic (dihydrosterculic) acid (DHSA) in their seeds (K. M. Schmid, U.S. Patent No. 5,936,139), similar to the levels reported upon their seed‐specific expression in transgenic Arabidopsis (Yu *et al*., 2011).

Engineering transgenic crop plants to accumulate high levels of mFA is a complex proposition in which its synthesis and subsequent incorporation into triacylglycerol (TAG) must be optimized while still retaining desirable growth and other agronomic traits. Our initial work on producing cyclopropane fatty acids in transgenic plants focused on proof‐of‐concept experiments in *Arabidopsis thaliana* (Yu *et al*., 2011, 2014). We previously screened CPS genes from bacteria and plants in Arabidopsis and determined that expression of the *E. coli* CPS (EcCPS) gene resulted in the highest level of CPA accumulation in a fatty acid desaturase 2 (FAD2, desaturation of 18 : 1–18 : 2)‐ and fatty acid elongase 1 (FAE1, elongation of 18 : 1–20 : 1)‐deficient (*fad2/fae1*) background that accumulates high levels of the 18 : 1, the CPS substrate (Yu *et al*., 2014). We next tested co‐expression of EcCPS with a number of acyltransferase genes and showed the *Sterculia foetida* lysophosphatidic acid acyltransferase (SfLPAT) provided the most significant increase in CPA accumulation. However, Arabidopsis seeds that accumulated more than 11% CPA exhibited strongly decreased germination and establishment, while seeds that accumulated more than 15% CPA showed little or no germination. Seed oil content was inversely correlated with CPA accumulation, in a similar manner to that reported for hydroxy‐ and conjugated‐FA accumulation (Bates *et al*., 2014; Cahoon *et al*., 2006).

In wild‐type Arabidopsis seeds, triacylglycerol (TAG) is primarily produced from phosphatidylcholine (PC)‐derived diacylglycerol (DAG), via *de novo* DAG to PC then to PC‐derived DAG, rather than from the conventional Kennedy pathway (Bates and Browse, 2011; Yang *et al*., 2017). Most of what we know about engineering mFA accumulation is derived from investigating hydroxy FA (HFA)‐accumulating transgenic plants (Horn *et al*., 2016). When the *Ricinus communis* fatty acid 12‐hydroxylase (RcFAH12) was expressed under control of a seed‐specific promoter in the *fae1* background (Bates and Browse, 2011), the resulting HFA‐CoA was efficiently incorporated into *de novo* DAG. However, *de novo* HFA‐DAG was not efficiently incorporated into membrane lipid or TAG. Instead, it was rapidly turned over, limiting the flux of HFA into PC, PC‐derived DAG and ultimately TAG. The HFA released from DAG turnover likely re‐entered glycerolipid metabolism and led to the observed down‐regulation of fatty acid synthesis that was attributed to post‐translational inhibition of plastid acetyl‐CoA carboxylase activity (Bates *et al*., 2014). The down‐regulation of fatty acid synthesis could be overcome by overexpressing the WRINKLED1 transcription factor in HFA‐accumulating lines (Adhikari *et al*., 2016).

To further our understanding of CPA metabolism in a crop plant, we chose the emerging oil crop camelina (*Camelina sativa* L.) because it is suitable for growth on marginal lands and is relatively easy to transform and its genome and transcriptome were recently reported (Kagale *et al*., 2014; Nguyen *et al*., 2013). Seed‐specific expression of the best two‐gene combination identified from our proof‐of‐principle experiments in Arabidopsis, that is EcCPS and SfLPAT in camelina, yielded close to 20% CPA accumulation in mature seeds. In contrast to our observations for Arabidopsis, transgenic camelina seeds germinated well and showed minor, if any, decreases in total seed oil content, suggesting camelina can tolerate higher levels of CPA accumulation with minimal impacts on normal metabolism and physiology. However, deficiencies in the transfer of CPA from PC to DAG and from DAG to TAG were identified. Mass spectrometry (MS) imaging of PC molecular species in seed sections revealed spatial segregation of mono‐ and di‐CPA‐PC, suggesting differential expression or utilization of genes encoding enzymes that transfer CPA from PC to TAG may contribute to the observed accumulation of CPA in PC. A model for the accumulation of substantial levels of CPA in camelina is proposed, and differences between CPA accumulation in camelina and Arabidopsis are discussed.

## Results

### Expression of CPSs in *Camelina sativa fad2/fae1*

**Figure 1:**
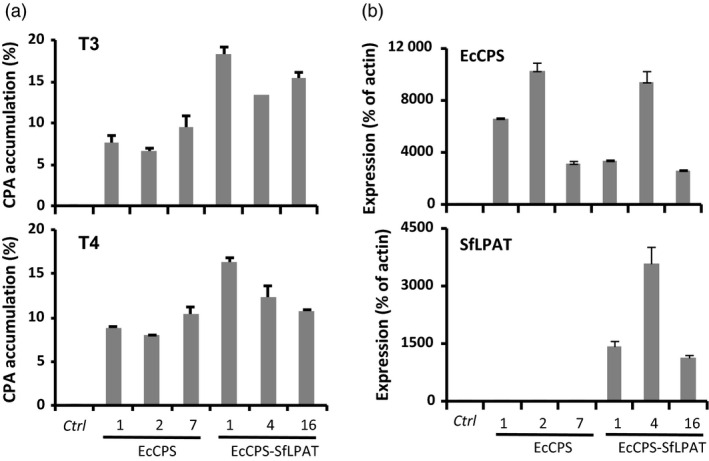
Cyclopropane fatty acid accumulation and transgene expression in T3 and T4 progeny upon the expression of indicated gene(s) in *fad2/fae1* plants. (a) Cyclopropane fatty acid accumulation in mature seeds of T3 and T4 progeny compared to camelina *fad2/fae1* (ctrl). Cyclopropane fatty acid (CPA) is expressed as a mol percentage of the total seed FA. Values represent the mean ± SD (*n *=* *3 pooled sets of 100 seeds). (b) EcCPS and SfLPAT expression in seeds of transgenic plants. qRT‐PCR analysis of EcCPS (top) and SfLPAT (bottom) expression levels in seeds of camelina *fad2/fae1* (ctrl) and three transgenic lines harbouring EcCPS or EcCPS and SfLPAT as indicated. The relative expression levels are reported relative to the expression of the Actin transcript. The values represent the mean ± SD of at least three biological replicates.

In *E. coli*, the primary substrate for CPS is monounsaturated FA‐esterified PE (Grogan and Cronan, 1997). In plants, 18 : 1‐esterified FA is also a substrate for FAD2 and FAE1 (Millar and Kunst, 1997; Okuley *et al*., 1994). FAD2 desaturates 18 : 1‐PC to form 18 : 2‐PC, and FAE1 elongates 18 : 1‐esterified FA to produce very long‐chain FA. Thus to reduce competing pathways for 18 : 1‐esterified FA precursors, we used a *fad2/fae1* camelina background that accumulates 70% 18 : 1 FA (Nguyen *et al*., 2013) generated through RNAi suppression of *FAD2* and *FAE1*. Four functional CPS‐encoding genes, including two from *Gossypium hirsutum* (upland cotton) (*GhCPS1, GhCPS2*), one from *Sterculia foetida* (*SfCPS*) and one from *E. coli* (*EcCPS*), were placed under the seed‐specific phaseolin promoter (Figure S1) and transformed into *fad2/fae1* camelina. Seeds from >10 independently transformed lines for each of the four constructs were analysed by gas chromatography (GC)–MS to determine their lipid composition. Expression of the three plant CPS‐encoding genes resulted in no detectable accumulation of CPA, but CPA was detected in lines expressing the *EcCPS*. T2 lines containing single insertion loci were identified and propagated to produce T3 homozygous seeds. In these T3 lines, CPA accumulated up to approximately 10% of the total seed FA (Figure 1a) in the three top producing lines.

### Co‐expression of EcCPS and SfLPAT in *fad2/fae1* camelina seeds

A second set of transgenic camelina was engineered in which the EcCPS gene was introduced into *fad2/fae1* camelina along with the SfLPAT, also under the control of the seed‐specific phaseolin promoter. T2 lines with single insertion loci and high CPA accumulation were planted to screen homozygous lines. In T3 camelina seeds, co‐expression of EcCPS and SfLPAT resulted in up to a 90% increase in CPA levels in the highest line accumulating CPA (Figure 1a line 1) compared to the highest line accumulating CPA expressing EcCPS alone (Figure 1a line 7). Mean levels of CPA in EcCPS‐expressing seeds were similar from T3 to T4 plants; however, seeds of EcCPS*‐*SfLPAT‐expressing T4 plants all showed slight decreases in CPA levels (Figure 1a) in lines 1, 4 and 16.

Quantitative RT‐PCR analysis of transgene expression in seeds confirmed EcCPS transcription in all the transgenic lines, and SfLPAT expression in the three EcCPS‐SfLPAT transgenic lines (Figure 1b). EcCPS expression was not elevated in the EcCPS‐SfLPAT lines relative to the EcCPS‐expressing lines, indicating the increased CPA accumulation in these lines resulted from the metabolic consequences of co‐expressing EcCPS‐SfLPAT. Variation in CPA accumulation in co‐expression lines did not directly correlate with the levels of SfLPAT expression.

### Seed weight and oil content of CPA‐accumulating camelina are similar to parental lines

**Figure 2:**
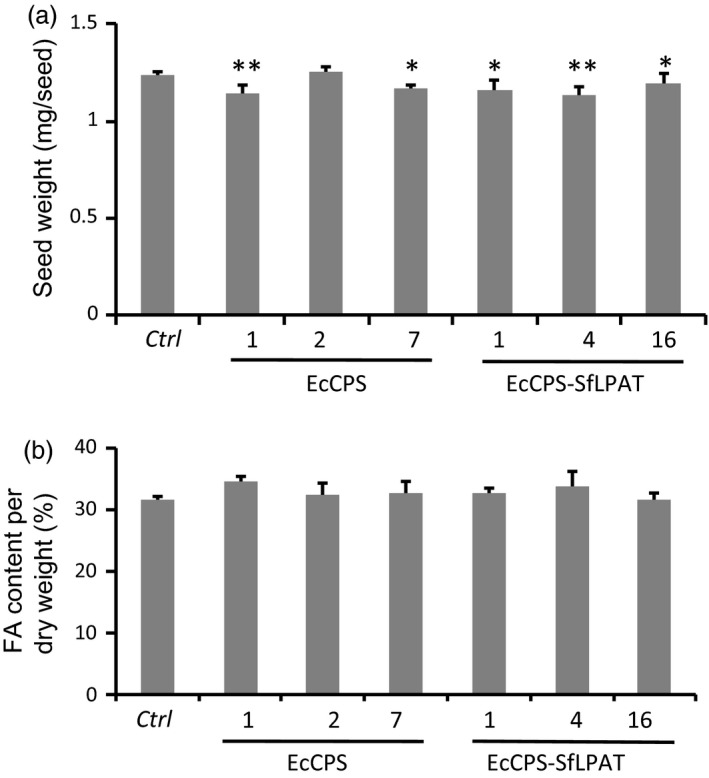
Seed weight and FA content in T4 camelina seeds. (a) Mean weight of transgenic seeds determined by five pooled sets of 100 seeds each. (b) Total fatty acid content in transgenic camelina seeds as a proportion of seed weight (panel A). Seed fatty acid content was quantified by GC of fatty acid methyl esters. Values represent means ± SD (*n *=* *3). **Student's *t*‐test *P *<* *0.01; *Student's *t*‐test, *P *<* *0.05.

Five of the six transgenic camelina lines showed a minor decrease (<10%) in the mean seed weight relative to the parental *fad2/fae1* seeds (Figure 2a). These fluctuations in seed weight were not directly correlated with CPA content as the EcCPS*‐*SfLPAT lines that accumulated the highest levels of CPA (line 1 in Figure 1a) showed only an approximately 7% decrease in seed weight and one of the lines, EcCPS line 2, showed no decrease in seed weight. Despite the minor reduction in seed weights, the homozygous T4 lines expressing EcCPS or co‐expressing SfLPAT and EcCPS showed no decrease in total fatty acid content although there was a negative correlation between seed size and total seed FA content (Figure 2b). Oil content as a per cent of seed dry weight was unaffected (Figure S2).

### EcCPS‐SfLPAT seeds are viable, but are mildly delayed in seedling establishment and development

In contrast to EcCPS‐SfLPAT‐expressing Arabidopsis in which seed germination was severely impaired (Yu *et al*., 2014), nearly 100% of the EcCPS‐SfLPAT T1 seeds germinated and developed into mature plants, although their germination and early growth on soil were slightly delayed relative to untransformed parental seeds and seeds expressing EcCPS alone (Figure S3a). Germination and development on half‐strength Murashige and Skoog medium supplemented with sucrose did not mitigate the delay (Figure S3b). Despite the observed differences in germination, EcCPS‐SfLPAT plants flowered normally (Figure S3c) and produced equivalent amount of seed relative to untransformed camelina grown at the same time.

Equivalent germination rates were observed for all lines. However, mild retardation of germination and growth was observed for T3 seeds with high CPA content from EcCPS lines 1, 2 and 7 and EcCPS‐SfLPAT lines 1, 4 and 16, suggesting that elevated CPA accumulation was responsible for the observed delay in germination. In contrast to results from Arabidopsis (Yu *et al*., 2014), in which seeds with CPA > 15% failed to germinate, camelina seeds with CPA > 15% showed no significant decrease in germination rates and the resulting seedlings once established developed normally.

### Distribution of CPA in polar and neutral lipids

**Figure 3:**
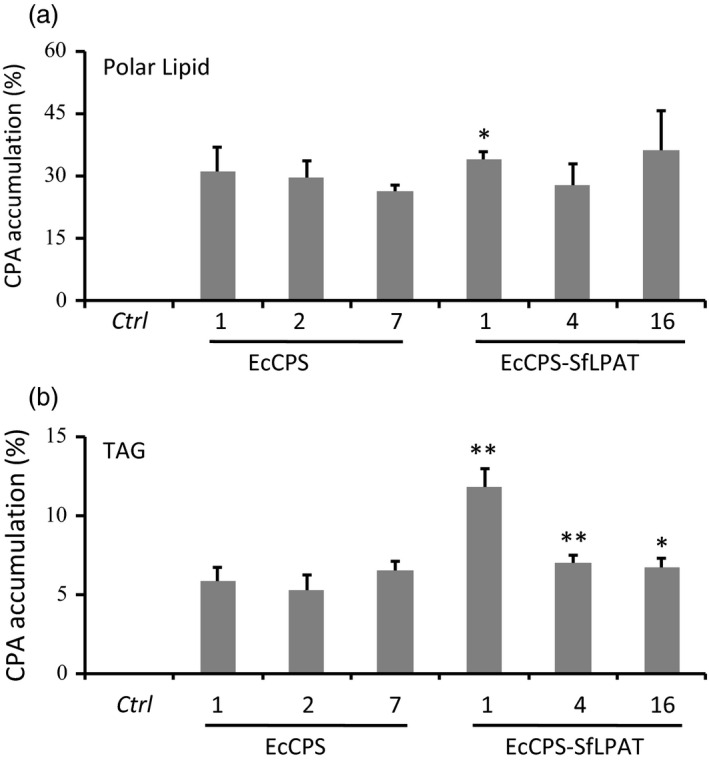
Cyclopropane fatty acid distribution in transgenic seeds. Cyclopropane fatty acid in polar lipids and TAG was expressed as a percentage of the total FA in (a). The percentage of CPA deposited in TAG was of total cyclopropane fatty acid (b). The values represent the mean and standard deviation of three replicates. **Student's *t*‐test *P *<* *0.01; *Student's *t*‐test, *P *<* *0.05.

EcCPS‐ and EcCPS‐SfLPAT‐expressing T4 seeds were analysed to determine whether the expression of SfLPAT influences the amount of CPA in polar lipids (e.g. primarily phosphatidylcholine (PC) and phosphatidylethanolamine (PE) in camelina seeds (Mansour *et al*., 2014). CPA accumulated to 26–31% in the polar lipid fraction of EcCPS‐expressing camelina lines (Figure 3 and Table 1), which was not significantly different (*P *>* *0.1) from that of the EcCPS‐ and SfLPAT‐co‐expressing lines that contained 28%–36% CPA. CPA accumulation in TAG increased significantly from 5% to 7% of dry weight (DW) to 7%–12% of DW when EcCPS was co‐expressed with SfLPAT (*P *<* *0.05; Figure 3b). In EcCPS line 1, CPA‐DAG (14%) was significantly lower than CPA‐polar lipids (31%) but also significantly higher than CPA‐TAG (6%) (Table 2). Furthermore, the increase in relative CPA‐TAG levels in EcCPS‐SfLPAT line 1 (12%) was associated with a significant increase (*P *<* *0.01) in CPA‐DAG (26%). These representative EcCPS and EcCPS‐SfLPAT lines were subjected to detailed lipidomics analyses.

**Table 1:** Fatty acid distribution in transgenic *fad2/fae1* camelina seeds

	16 : 0%	18 : 0%	18 : 1%	18 : 2%	18 : 3%	20C%	CPA%
Polar Lipid							
* fad2/fae1*	21.6 ± 4.2	23.0 ± 3.4	49.0 ± 2.8	4.2 ± 3.0	2.1 ± 1.8	nd	nd
EcCPS1	18.2 ± 2.5	21.4 ± 2.5	21.7 ± 3.5	3.9 ± 2.9	3.7 ± 2.6	nd	31.1 ± 5.7
EcCPS2	16.7 ± 2.0	18.2 ± 1.6	26.6 ± 2.0	3.9 ± 2.8	3.5 ± 2.5	1.3 ± 1.9	29.7 ± 3.9
EcCPS7	17.4 ± 2.2	21.0 ± 3.2	25.7 ± 3.1	4.9 ± 0.5	3.4 ± 1.4	1.4 ± 1.8	26.2 ± 1.6
EcCPS‐SfLPAT1	19.3 ± 3.0	15.6 ± 2.7	22.3 ± 2.3	5.4 ± 0.5	3.1 ± 0.7	nd	34.1 ± 1.5
EcCPS‐SfLPAT4	18.8 ± 1.7	15.3 ± 1.9	29.8 ± 1.6	3.7 ± 2.7	3.3 ± 0.8	1.4 ± 1.9	27.7 ± 5.0
EcCPS‐SfLPAT16	19.4 ± 1.7	20.0 ± 2.8	18.4 ± 3.1	4.2 ± 3.0	1.8 ± 0.3	nd	36.1 ± 9.5
TAG							
*fad2/fae1*	6.4 ± 0.3	3.0 ± 0.1	72.9 ± 2.5	5.6 ± 0.8	7.6 ± 1.1	4.6 ± 0.5	nd
EcCPS1	5.6 ± 0.2	3.2 ± 0.1	68.0 ± 2.6	4.2 ± 0.4	6.9 ± 0.8	6.3 ± 1.1	5.9 ± 0.8
EcCPS2	6.1 ± 0.3	3.2 ± 0.3	67.1 ± 3.3	4.8 ± 0.5	7.9 ± 0.9	5.6 ± 1.3	5.3 ± 0.9
EcCPS7	5.8 ± 0.2	4.4 ± 1.5	63.9 ± 2.8	4.3 ± 0.2	7.3 ± 1.2	7.4 ± 1.1	6.5 ± 0.6
EcCPS‐SfLPAT1	7.7 ± 0.4	3.4 ± 0.1	56.7 ± 1.9	4.6 ± 0.2	9.3 ± 2.1	6.2 ± 0.3	11.8 ± 1.2
EcCPS‐SfLPAT4	6.8 ± 0.1	2.8 ± 0.1	63.5 ± 2.0	5.6 ± 0.3	9.5 ± 1.2	4.8 ± 0.9	7.0 ± 0.5
EcCPS‐SfLPAT16	7.1 ± 0.2	3.0 ± 0.1	61.1 ± 2.1	5.5 ± 0.4	10.1 ± 1.7	6.5 ± 0.4	6.7 ± 0.6

Values represent mean weight per cent ± SD (*n *=* *3); nd, not detectable; 20C represents the sum of 20 : 0 and 20 : 1.

**Table 2:** Fatty acid composition of DAG in transgenic *fad2/fae1* camelina seeds

	16 : 0%	18 : 0%	18 : 1%	18 : 2%	18 : 3%	20 : 1%	CPA%
*fad2/fae1*	5.0 ± 0.2	2.8 ± 0.1	69.6 ± 0.5	7.9 ± 0.1	11.7 ± 0.4	2.5 ± 0.3	nd
EcCPS1	5.7 ± 0.2	3.7 ± 0.0	57.8 ± 0.1	5.9 ± 0.8	9.3 ± 0.3	3.4 ± 0.1	14.3 ± 0.2
EcCPS‐SfLPAT1	6.0 ± 0.1	2.3 ± 0.2	42.6 ± 1.2	7.2 ± 0.1	13.4 ± 0.2	2.4 ± 0.1	26.0 ± 0.9

Values represent mean weight per cent ± SD (*n *=* *3); nd, not detectable.

### Compositional and stereospecific analysis of CPA in PC, DAG and TAG

Plant SfCPS has been reported to act primarily on 18 : 1 esterified to the *sn*‐1 position of PC, whereas the EcCPS was reported to mainly act on 18 : 1 esterified to the *sn‐*2 position of PE in *E. coli* (because the *sn‐1* position is mostly occupied by saturated FA) (Bao *et al*., 2003; Hildebrand and Law, 1964). To determine whether the *in vivo* substrate of EcCPS is PC and/or PE when expressed in camelina, we first separated the PC and PE fractions from the polar lipid fraction and analysed their composition. In *fad2/fae1* camelina seeds, 18 : 1 is present at 71% in PC and 59% in PE (Table 3) with PE containing a higher percentage of saturated fatty acids (i.e. 16 : 0, 18 : 0) than PC. Transgenic lines expressing EcCPS showed a decrease in 18 : 1 and increase in CPA, which constitute 62% and 68% of the fatty acid species in PC and PE, respectively. Co‐expression of SfLPAT with EcCPS did not significantly increase the relative amount of CPA in PC or PE (Table 3).

**Table 3:** Fatty acid composition of PC and PE in transgenic *fad2/fae1* camelina seeds

	16 : 0%	18 : 0%	18 : 1%	18 : 2%	18 : 3%	CPA%
PC						
* fad2/fae1*	15.5 ± 1.1	9.3 ± 0.4	72.2 ± 0.8	3.0 ± 0.9	nd	nd
EcCPS1	10.9 ± 1.0	7.5 ± 2.0	18.9 ± 7.5	1.1 ± 0.5	nd	61.6 ± 6.0
EcCPS‐SfLPAT1	14.7 ± 1.1	12.3 ± 3.1	7.9 ± 1.8	1.0 ± 0.2	nd	64.8 ± 1.8
PE						
* fad2/fae1*	22.1 ± 2.3	9.7 ± 2.0	59.3 ± 1.1	6.1 ± 0.3	2.9 ± 0.4	nd
EcCPS1	12.8 ± 0.8	10.2 ± 2.0	7.8 ± 1.1	1.4 ± 0.2	nd	67.8 ± 0.9
EcCPS‐SfLPAT1	15.1 ± 1.5	9.3 ± 1.5	7.4 ± 0.4	0.5 ± 0.7	nd	67.7 ± 2.0

Values represent mean weight per cent ± SD (*n *=* *3); nd, not detectable.

Positional analysis of PC in parental *fad2/fae1* camelina shows the 18 : 1 substrate accumulates to 62% and 83% in the *sn*‐1 and *sn*‐2 positions of PC, respectively (Table 4). Upon EcCPS expression, *sn*‐1 18 : 1 was reduced 10‐fold (relative to *fad2/fae1*) to 6% with a substantial increase in CPA accumulating up to 74%. In contrast, at the *sn*‐2 position, 18 : 1 was reduced less than threefold (relative to *fad2/fae1*) to 31% with CPA only accumulating up to 49%. That the 18 : 1 substrate is higher at the *sn*‐2 position of both PC and PE (Table 5), but relatively higher levels of CPA accumulation at the *sn*‐1 position are consistent with the EcCPS acting at *sn*‐1 and to a lesser extent at the *sn*‐2 positions when expressed in Arabidopsis (Yu *et al*., 2014). The approximate 1.5‐fold excess of CPA accumulation at *sn*‐1 relative to *sn*‐2 seen in PC and PE for the EcCPS lines was abrogated upon co‐expression of SfLPAT with EcCPS which resulted in a more equal distribution of CPA between *sn*‐1 and *sn*‐2.

**Table 4:** Fatty acid composition of *sn*‐1 and *sn*‐2 positions of PC from transgenic *fad2/fae1* camelina seeds

	16 : 0%	18 : 0%	18 : 1%	18 : 2%	CPA%
*fad2/fae1*					
*sn‐1*	29.6 ± 1.5	6.1 ± 0.9	62.3 ± 1.5	1.9 ± 0.4	nd
*sn*‐2	0.3 ± 0.3	12.7 ± 0.4	82.9 ± 2.4	4.0 ± 2.4	nd
EcCPS 1					
*sn‐1*	13.8 ± 0.6	4.6 ± 1.4	6.4 ± 2.8	0.4 ± 0.3	74.4 ± 1.5
*sn*‐2	8.0 ± 1.6	10.4 ± 3.4	31.3 ± 12.2	1.7 ± 0.7	48.9 ± 10.6
EcCPS‐SfLPAT 1					
*sn‐1*	20.6 ± 1.4	8.8 ± 2.2	2.9 ± 1.9	0.2 ± 0.3	67.5 ± 1.2
*sn*‐2	8.9 ± 2.5	15.7 ± 7.7	12.9 ± 2.6	1.7 ± 0.5	62.1 ± 3.9

Values represent mean weight per cent ± SD *(n *=* *3); nd, not detectable.

**Table 5:** Fatty acid composition of *sn*‐1 and *sn*‐2 positions of PE from transgenic *fad2/fae1* camelina seeds

	16 : 0%	18 : 0%	18 : 1%	18 : 2%	18 : 3%	CPA%
*fad2/fae1*						
*sn‐1*	40.4 ± 0.7	4.7 ± 0.8	48.3 ± 2.1	6.6 ± 0.7	nd	nd
*sn*‐2	3.7 ± 0.7	14.6 ± 3.6	70.3 ± 1.0	5.7 ± 1.2	5.7 ± 0.8	nd
EcCPS 1						
*sn‐1*	12.7 ± 0.3	1.8 ± 0.1	1.4 ± 0.2	0.0 ± 0.0	nd	84.0 ± 0.2
*sn*‐2	12.9 ± 1.9	18.6 ± 4.2	14.1 ± 2.0	2.8 ± 0.5	nd	51.6 ± 1.9
EcCPS‐SfLPAT 1						
*sn‐1*	27.0 ± 0.3	3.8 ± 0.2	2.7 ± 0.1	0.0 ± 0.0	nd	66.5 ± 0.5
*sn*‐2	3.2 ± 3.2	14..8 ± 3.0	12.1 ± 0.8	1.0 ± 1.4	nd	68.9 ± 4.3

Values represent mean weight per cent ± SD (*n *=* *3); nd, not detectable.

A similar distribution of 18 : 1 at *sn*‐1 and *sn*‐2 positions of DAG relative to PC was observed in the *fad2/fae1* lines (Table 6). However, unlike in PC, EcCPS lines showed a more uniform distribution of CPA with a slight enrichment in the *sn*‐2 position (16% versus 13%). Also despite having similar CPA amounts, the relative proportion of *sn*‐2 18 : 1 was much lower relative to parental lines. While the proportion of CPA increased in both positions in EcCPS‐SfLPAT1 lines, the proportional increase was much higher in the *sn*‐2 position (twofold versus 1.5‐fold) relative to EcCPS lines alone, similar to the *sn*‐2 CPA enrichment observed in PC. Total CPA content in TAG approximately doubled from 6% in the EcCPS line to 12% in the EcCPS‐SfLPAT line even though they were both enriched in CPA at the *sn‐*1,3 position relative to *sn*‐2 positions of TAG (Table 7). This distribution was more similar to PC than DAG.

**Table 6:** Fatty acid composition of *sn*‐1 and *sn*‐2 positions of DAG from transgenic camelina seeds

	16 : 0%	18 : 0%	18 : 1%	18 : 2%	18 : 3%	20 : 1%	CPA%
*fad2/fae1*							
*sn*‐1	8.5 ± 0.5	3.0 ± 0.6	58.5 ± 0.6	9.9 ± 0.8	15.1 ± 0.8	5.1 ± 0.5	nd
*sn*‐2	2.5 ± 0.1	2.5 ± 0.5	80.6 ± 0.6	5.9 ± 1.0	8.4 ± 0.1	nd	nd
EcCPS 1							
*sn*‐1	7.3 ± 1.0	2.7 ± 0.6	55.9 ± 2.5	5.5 ± 1.8	9.0 ± 0.9	6.8 ± 0.2	12.8 ± 0.2
*sn*‐2	4.0 ± 0.7	4.6 ± 0.5	59.6 ± 2.4	6.3 ± 1.1	9.7 ± 0.3	nd	15.8 ± 0.5
EcCPS‐SfLPAT 1							
*sn*‐1	8.5 ± 0.4	1.5 ± 0.2	35.6 ± 1.9	9.5 ± 0.4	19.7 ± 0.5	4.9 ± 0.1	20.3 ± 1.7
*sn*‐2	3.4 ± 0.2	3.1 ± 0.2	49.6 ± 0.5	5.0 ± 0.1	7.1 ± 0.2	nd	31.8 ± 0.1

Values represent mean weight per cent ± SD (*n* = 3); nd, not detectable.

**Table 7:** Fatty acid composition of *sn* position of TAG from transgenic camelina seeds

	16 : 0%	18 : 0%	18 : 1%	18 : 2%	18 : 3%	20 : 0%	20 : 1%	22 : 1%	CPA%
*fad2/fae1*									
*sn*‐1,3	11.0 ± 0.3	6.2 ± 0.4	51.7 ± 1.4	7.9 ± 0.3	12.3 ± 0.3	1.3 ± 0.1	8.4 ± 0.5	0.8 ± 0.0	nd
*sn*‐2	0.0 ± 0.0	0.0 ± 0.0	66.1 ± 1.1	9.0 ± 0.5	11.5 ± 0.6	nd	nd	nd	nd
EcCPS 1									
*sn*‐1,3	9.6 ± 0.4	6.0 ± 1.2	50.5 ± 3.6	6.0 ± 0.6	11.1 ± 0.7	1.3 ± 0.1	10.1 ± 1.2	0.9 ± 0.6	7.2 ± 0.7
*sn*‐2	0.0 ± 0.0	0.0 ± 0.0	64.6 ± 1.0	7.3 ± 0.9	9.8 ± 0.5	nd	nd	nd	3.0 ± 0.6
EcCPS‐SfLPAT 1									
*sn*‐1,3	11.9 ± 0.4	6.8 ± 0.1	41.8 ± 0.4	7.2 ± 0.6	15.0 ± 0.4	1.4 ± 0.0	8.8 ± 1.0	0.8 ± 0.0	13.8 ± 1.3
*sn*‐2	0.0 ± 0.0	0.4 ± 0.3	63.0 ± 0.7	7.9 ± 0.3	9.9 ± 0.6	nd	nd	nd	6.0 ± 0.8

Values represent mean weight per cent ± SD (*n* = 3); nd, not detectable.

### Spatial distribution and quantification of lipid species in camelina seeds by MALDI/MSI

**Figure 4:**
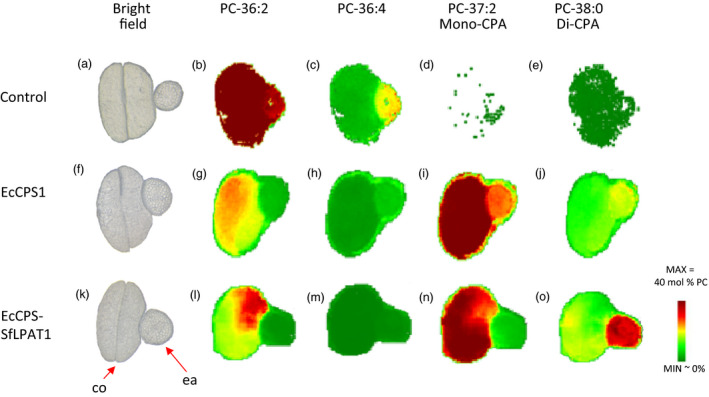
MSI imaging of selected phosphatidylcholine (PC) molecular species in camelina seeds. (a), (f) and (k). Bright‐field cross‐sectional image. Abbreviations: co, cotyledons; ea, embryonic axis. (b–e), (g–j) and (l–o). Relative distribution profiles of selected PC molecular species. [Colour figure can be viewed at wileyonlinelibrary.com]

Imaging of lipids in seed sections by mass spectrometry was previously established for camelina by our group (Horn *et al*., 2013). Here, MALDI‐MS imaging was similarly used to localize molecular species of PC in sections of transgenic camelina seeds as a means of localizing the CPA‐containing PC. The results showed that in control *fad2/fae1* seeds, the majority of PC species are di‐18 : 1‐PC (i.e. in which 18 : 1 occupies both *sn*‐1 and *sn*‐2 positions; Figure 4b), which was nearly evenly distributed spatially throughout the embryo with respect to the embryonic axis and cotyledon (Figure 4 and Table 8). Expression of EcCPS converted the di‐18 : 1‐PC to both mono‐CPA‐PC (18 : 1–19 : 0 CPA‐PC or 19 : 0 CPA‐18 : 1‐PC; Figure 4i and Table 8) and di‐CPA‐PC (in which CPA occupies both *sn*‐1 and *sn*‐2 position; Figure 4j and Table 8). Mono‐CPA‐PC primarily accumulated in the cotyledon. Co‐expression of SfLPAT increased the fraction of di‐CPA‐PC in the total PC from 14% to 19% (Table 8). Interestingly, the di‐CPA‐PC predominantly accumulates in the embryonic axis. To test whether this was related to levels of transgene expression, we performed expression analysis which showed EcCPS and SfLPAT were equally expressed in both tissues (Figure S4). The expression level of endogenous LPAT was previously reported to be low and equivalent for camelina embryo axis and cotyledon tissues (Jiao *et al*., 2013). These data suggest that the transfer of CPA from PC to TAG is inefficient and may be more pronounced in the embryonic axis than in the cotyledon.

**Table 8:** Relative quantification of individual PC species in MALDI/MSI cross sections

	*fad2/fae1*	EcCPS1	EcCPS‐SfLPAT1
34 : 2	1.2 ± 0.3	1.4 ± 0.3	2.2 ± 0.1
34 : 1	14.9 ± 2.4	6.5 ± 0.1	5.7 ± 0.3
35 : 1‐CPA	0.1 ± 0.0	1.0 ± 0.1	0.7 ± 0.0
35 : 0‐CPA	0.1 ± 0.1	3.7 ± 0.2	5.3 ± 0.3
36 : 4	6.8 ± 1.1	3.0 ± 0.5	2.1 ± 0.2
36 : 3	5.6 ± 0.3	2.5 ± 0.2	2.4 ± 0.1
36 : 2	65.2 ± 4.2	17.4 ± 1.1	16.2 ± 0.9
37 : 3‐CPA	0.0 ± 0.0	4.6 ± 0.9	4.6 ± 0.3
37 : 2‐CPA	0.0 ± 0.0	3.5 ± 0.6	5.2 ± 0.2
37 : 1‐CPA	0.4 ± 0.1	38.1 ± 1.9	30.3 ± 0.3
38 : 2‐CPA‐CPA/38 : 4	0.0 ± 0.0	0.3 ± 0.1	1.7 ± 0.2
38 : 0‐CPA‐CPA	0.5 ± 0.1	14.2 ± 0.9	19.4 ± 0.3
38 : 1	0.0 ± 0.0	0.7 ± 0.1	0.8 ± 0.2
Total CPA‐containing PC	1.1 ± 0.2	65.8 ± 1.2	67.7 ± 0.7

Values represent mean mol per cent ± SD (*n* = 3).

### Fatty acid distribution in cotyledon and embryo axis

To independently substantiate the MALDI results, analyses of total fatty acids from excised embryo axis and cotyledon tissues were performed. The embryo axis contained higher levels of CPA than the cotyledons (Table 9), validating the MALDI‐MS imaging results. In both transgenic lines investigated, CPA levels are approximately twofold higher in the embryo axis than in the cotyledon. We noted that both 18 : 2 (∆9,12) and 18 : 2 (∆9,15) were identified in the cotyledons of camelina, each representing approximately 2%–3% of the total fatty acids in the cotyledons of all the transformed and nontransformed camelina seeds, while only 18 : 2 (∆9, 12) was detected in the embryo axis of *fad2/fae1* camelina and EcCPS‐expressing lines.

**Table 9:** Fatty acid distribution in embryo axis and cotyledon

	16 : 0%	18 : 0%	18 : 1%	18 : 2^a^%	18 : 2^b^%	18 : 3%	20 : 1%	CPA%
Cotyledon								
*fad2/fae1*	6.8 ± 0.5	7.2 ± 1.6	68 ± 1.0	2.1 ± 0.1	2.7 ± 0.1	8.7 ± 0.4	4.3 ± 0.6	nd
EcCPS1	6.5 ± 0.7	6.1 ± 1.7	55.6 ± 7.8	2.5 ± 0.2	1.8 ± 0.3	15.4 ± 8.8	3.8 ± 0.8	8.8 ± 2.6
EcCPS1‐SfLPAT	8.0 ± 0.2	4.7 ± 0.4	49.3 ± 1.6	2.9 ± 0.1	2.3 ± 0.1	20.0 ± 3.1	3.4 ± 0.2	9.9 ± 2.2
Embryo axis								
*fad2/fae1*	12.2 ± 0.3	9.9 ± 0.3	49.4 ± 1.2	6.2 ± 0.1	nd	18.0 ± 0.7	4.3 ± 0.2	nd
EcCPS1	9.8 ± 0.3	5.2 ± 0.2	50 ± 1.3	4.5 ± 0.2	nd	10.6 ± 1.2	4.3 ± 0.3	15.6 ± 1.0
EcCPS‐SfLPAT1	10.4 ± 1.0	5.4 ± 0.7	38.7 ± 3.7	5.9 ± 0.5	0.9 ± 0.2	15.4 ± 1.8	3.3 ± 0.1	19.5 ± 0.6

Values represent mean weight per cent ± SD (*n* = 3); nd, not detectable.

a 18 : 2 (∆9,12).

b 18 : 2 (∆9,15).

### MS analysis of CPA‐DAG

**Figure 5:**
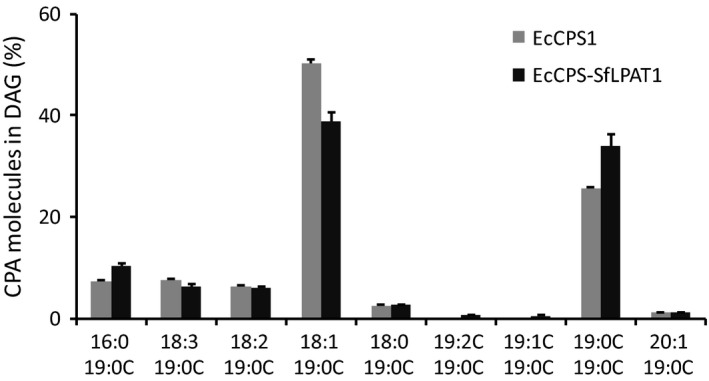
MS analysis of CPA‐containing DAG. CPA‐containing DAGs were detected by neutral loss of CPA (*m/z* 313.2, [M+NH_3_]) with electrospray ionization source under positive ion mode by direct infusion. The values represent the mean and standard deviation of three replicates.

As discussed above, co‐expression of EcCPS with SfLPAT increased di‐CPA‐PC from 22% to 29% of CPA‐PC. To test whether this affects its conversion to DAG, CPA‐containing DAG molecular species were analysed through direct infusion‐electrospray ionization (ESI)–MS. In the presence of SfLPAT, the proportion of di‐CPA‐DAG relative to the total CPA‐DAG increased from 25% to 35% of the total DAG (Figure 5), indicating that di‐CPA‐PC is converted preferentially over mono‐CPA‐PC to di‐CPA‐DAG or that di‐CPA‐DAG is less well utilized relative to mono‐CPA‐DAG.

### 19 : 0 CPA can be elongated and desaturated in camelina seeds

**Figure 6:**
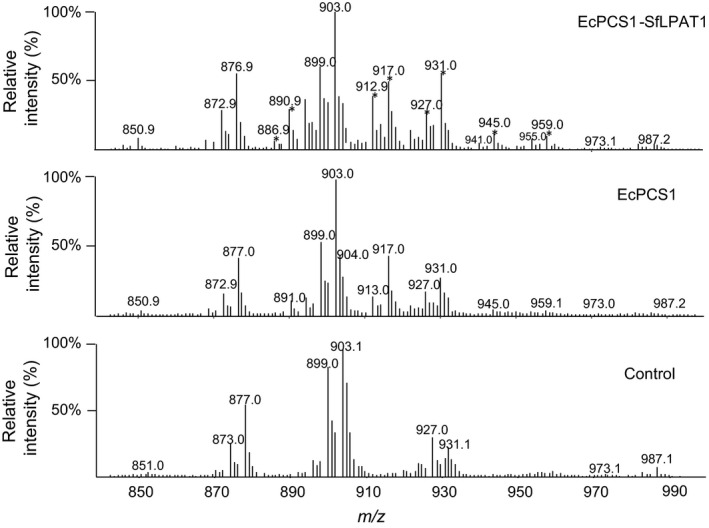
TAG profiles: Q1 scans showing M+NH4 intact TAG species. Abundant M+NH4 ions in the *fad2/fae1* sample are 927.0 (18 : 3/18 : 1/20 : 1 or 56 : 5), 903.1 (tri‐18 : 1), 899.0 (18 : 3/18 : 1/18 : 1 or 54 : 5), 877.0 (16 : 0/18 : 1/18 : 1) and 873.0 (16 : 0/18 : 1/18 : 3 or 52 : 4). *TAG species with odd‐numbered fatty acid species (c19 : 0 primarily but may also contain c21 : 0 and c23 : 0 (elongation products of c19 : 0 DHSA).

Q1 scanning of total neutral lipid extracts showed novel c19 : 0‐containing M+NH_4_ TAG species in EcCPS1 and EcCPS*‐*SfLPAT1 (Figure 6). Lipidomics analyses through LC/MS also showed that a small amount of 19 : 0 CPA was elongated to 21 : 0 and 23 : 0 (Figure S5). Detection of the neutral loss (NL) ion *m/z* 341.2 corresponding to 21 : 0 CPA confirms that 19 : 0 CPA can be elongated to 21 : 0 CPA and incorporated into TAG species with *m/z* 918.8 (16 : 0/18 : 1/21 : 0 with *sn* position nonstereospecific in these designations), *m/z* 941.0 (18 : 3/18 : 3/21 : 0), *m/z* 944.9 (18 : 1/18 : 1/21 : 0), *m/z* 959.0 (18 : 1/19 : 0/21 : 0) and *m/z* 973.1 (19 : 0/19 : 0/21 : 0). Neutral loss spectra for *m/z* 944.9, *m/z* 959.0 and *m/z* 973.0 TAG confirmed the presence of 21 : 0 CPA‐containing DAGs (Figure S5a). Detection of the NL ion at *m/z* 369.0 indicates that TAG species *m/z* 973.0 and 987.0 contain 23 : 0 CPA (i.e. elongation products of 19 : 0 CPA). A small amount of 18 : 1/18 : 1/23 : 0 was present in *m/z* 973.0 TAG, and a small amount of TAG with *m/z* 986.8 representing 18 : 1/19 : 0/23 : 0 was detected (Figure 3b). Very long‐chain CPA (21 : 0) was also detected in the TAG of EcCPS‐SfLPAT lines by the use of GC/MS, but it co‐eluted with 22 : 1 (Figure S6). Trace amounts of desaturated 19 : 1 CPA were also detected by GC‐MS (Figure S6).

## Discussion

Important findings arise from this work: (i) two major bottlenecks were identified in the accumulation of CPA‐TAG, namely the conversion of CPA‐PC to CPA‐DAG and the conversion of CPA‐DAG to CPA‐TAG. (ii) Co‐expression of EcCPS and SfLPAT resulted in accumulation of >15% of CPA in the seeds of the oil crop camelina. (iii) Camelina seeds with increased CPA accumulation germinated similarly to wild‐type seeds giving rise to plants that were visually indistinguishable from wild type. (iv) Expression of EcCPS in transgenic seed led to the accumulation of CPA in both PC and PE, enriched at the *sn‐1* positions. (v) Co‐expression of EcCPS with SfLPAT increased di‐CPA‐PC accumulation relative to mono‐CPA‐PC resulting in enhanced CPA accumulation in DAG and subsequently in TAG. (vi) CPA content is distributed heterogeneously within individual seed embryos, with twofold excess in the embryonic axis relative to the cotyledon, and MS imaging showed that mono‐CPA is primarily distributed in the cotyledon, while di‐CPA‐PC is mainly present in the embryonic axis. (vii) Finally, lipidomics analysis shows that 19 : 0 CPA can be both elongated and desaturated.

### The expression of plant CPS genes resulted in no detectable CPA accumulation

We previously reported that heterologous expression of genes encoding CPS from cotton and *Sterculia foetida* in Arabidopsis resulted in the accumulation of up to 1% CPA, and expression of *E. coli* CPS resulted in the accumulation an average of 5% CPA in T1 seeds (Yu *et al*., 2014). Here, we report that expression of the same constructs in camelina resulted in nondetectable levels of CPA from the plant genes but similar levels for the *E. coli* gene. Because the goal of this work was to optimize the accumulation of CPA in seeds of a crop plant, we did not further investigate the apparently paradoxical finding that expression of a bacterial CPS results in a stronger phenotype than expression of several plant CPSs. There are many possible explanations for this observation; for instance, it is possible that either the CPS mRNA or the CPS enzyme is degraded in seed tissues. Alternatively, an additional oxidase‐like domain at the N‐terminus of the plant enzymes might constitute a second activity that could further metabolize CPA to an as yet unidentified product. Another possibility is that the plant enzymes are regulated differently than the bacterial enzyme and that upon heterologous expression in seed tissue, either the lack of a proteinaceous or allosteric positive regulator or the presence of a negative regulator restricts its activity. Further study is needed to distinguish between these possibilities.

### Co‐expression of EcCPS and SfLPAT can cause the accumulation of >15% CPA in camelina seeds which germinate normally

To engineer the production of CPA‐containing TAG in the crop plant camelina, we tested the expression of both plant and microbial CPS enzymes under the control of the seed‐specific phaseolin promoter. EcCPS outperformed all of the plant orthologs, yielding up to approximately 10% CPA in transgenic camelina seeds under which conditions no CPA was detected upon expression of the plant orthologs. These data are consistent with our previous results from Arabidopsis in that the CPS from *E. coli* outperformed plant CPS enzymes when heterologously expressed in plants (Yu *et al*., 2014).

In *E. coli*, EcCPS converts monoenes on PE to CPA (Bao *et al*., 2003; Hildebrand and Law, 1964). That *E. coli* lacks PC raises the question as to whether PE, PC or both are substrates when EcCPS is expressed in plants. In this work, we show that when EcCPS is expressed in camelina, oleic acid decreases and CPA increases to >65% on both PE and PC, suggesting CPS recognizes both substrates. While the data presented herein cannot formally discount the possibility that all the CPA‐PC is derived from CPA‐PE, the observation that in camelina PE is a very minor component relative to PC (Mansour *et al*., 2014) would seem to argue against this possibility.

While co‐expression of SfLPAT with EcCPS in Arabidopsis seeds resulted in the accumulation of higher levels of CPA than the expression of EcCPS alone, increased CPA accumulation was accompanied by a significant 18% decrease in total fatty acid content (Yu *et al*., 2014). Additionally, seeds containing more than 11% CPA exhibited dramatically decreased germination, with no observed germination of seeds containing more than 15% CPA. Similar reductions in total fatty acid content and germination rates were reported upon the expression of an oleate hydroxylase in Arabidopsis (Bates and Browse, 2011; van Erp *et al*., 2011) and the *Momordica* conjugase gene in soya bean (Cahoon *et al*., 2006; van Erp *et al*., 2011). However, co‐expression of EcCPS along with SfLPAT in camelina resulted in close to 20% CPA accumulation in seeds that exhibited total fatty acid content and germination rates similar to those of the parental *fad2/fae1* line, suggesting that the reduction in total fatty acid content to below a threshold level needed for germination is responsible for the reduced germination phenotype of CPA‐accumulating Arabidopsis. That the reduced germination of ricinoleic acid‐containing seeds can be reversed upon co‐expression of WRINKLED1 is consistent with this view (Adhikari *et al*., 2016).

### Bottlenecks for CPA‐TAG accumulation are different from those associated with hydroxy fatty acids

In Arabidopsis, most mFA in TAG originates from the PC pool (Bates and Browse, 2011). The expression of EcCPS either alone or with SfLPAT in camelina results in CPA accumulation to greater than 65% on PC. However, upon the co‐expression of SfLPAT, the percentage of CPA in DAG increases from 14 to 26%, nearly doubling the amount of CPA at both *sn*‐1 and *sn*‐2 positions, and the level of CPA‐TAG also approximately doubles from around 6%–12%. PC can be converted to DAG by two routes, one via phosphatidylcholine : diacylglycerol cholinephosphotransferase (PDCT) and the other via the release of CPA from PC which subsequently enters the CoA pool and is incorporated into TAG via the Kennedy pathway. The main bottlenecks are likely the metabolism of CPA‐PC and CPA‐PC‐derived DAG, which differ from those reported for ricinoleic acid in Arabidopsis in which the main bottleneck is the conversion of *de novo* DAG to PC (Bates *et al*., 2014). Promising approaches to overcoming these bottlenecks in CPA accumulation include expressing additional source plant acyltransferase genes in EcCPS‐SfLPAT camelina lines. To improve the metabolism of CPA from CPA‐PC to DAG and on to TAG, a phospholipid diacylglycerol acyltransferase (PDAT) with high specificity towards CPA‐PC and DAG and/or a more active PDCT enzyme that does not discriminate between CPA and the normal complement of fatty acids such as those from source tissues such as lychee seeds (Gontier *et al*., 2000) are appealing candidates. To mediate the conversion of CPA‐DAG to CPA‐TAG, DGAT and/or PDAT enzymes with high selectivity for CPA‐DAG, or a more highly active enzyme with broad specificity, would be required. Recent reports documenting improvement in the stability/activity of acyltransferases represent a complementary approach that could contribute to mFA accumulation (Greer *et al*., 2015; Roesler *et al*., 2016).

Di‐CPA species were identified in both EcCPS transgenics and EcCPS‐SfLPAT transgenics by mass spectrometry imaging of PC, indicating that both camelina LPAT and *Sterculia* LPAT can utilize CPA‐CoA to acylate CPA‐LPA. That SfLPAT did not alter the CPA content in PC but increased di‐CPA‐PC from 22% to 29% confirms that SfLPAT has a preference for CPA‐LPA and CPA‐CoA that results in a CPA acyl exchange cycle as previously reported (Yu *et al*., 2014). This preference is similar to that seen for castor bean LPAT2, which prefers ricinoleoyl‐CoA over other fatty acid thioesters when ricinoleoyl‐LPA is used as the acceptor (Arroyo‐Caro *et al*., 2013), thereby enhancing ricinoleic acid at the *sn*‐2 position of TAGs when expressed in *Lesquerella fendleri* seed (Chen *et al*., 2016). In the presence of SfLPAT, the proportion of di‐CPA‐DAG increased from 25% to 35% of total CPA‐DAG implying that di‐CPA‐PC is preferred over mono‐CPA‐PC for conversion to di‐CPA‐DAG. As CPA can accumulate to over 65% in PC, the conversion of *de novo* DAG to PC appears not to limit CPA synthesis, in contrast to Bates’ suggestion that the *de novo* DAG to PC step is the bottleneck for mFA synthesis, in particular hydroxy FA synthesis (Bates *et al*., 2014). Upon co‐expression of EcCPS and SfLPAT PC contained 65% of CPA, DAG contained 26% of CPA and TAG only 12%, demonstrating that the utilization of CPA‐PC and CPA‐DAG are the major bottlenecks.

### Engineering metabolism to further increase cyclic FA accumulation

**Figure 7:**
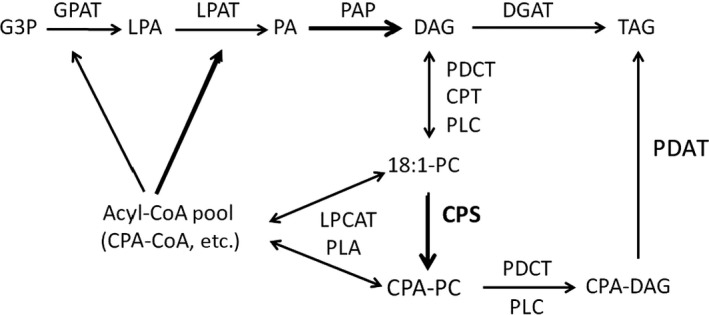
Plant triacylglycerol biosynthesis network Acyl editing can provide PC‐modified FAs for *de novo* DAG/TAG synthesis. Substrate abbreviations: DAG, diacylglycerol; G3P, glycerol‐3‐phosphate; LPA, lyso‐phosphatidic acid; LPC, lyso‐phosphatidylcholine; mFA, modified FA; PA, phosphatidic acid; PC, phosphatidylcholine; TAG, triacylglycerol; FAS, Fatty acid synthase; CPT, CDP‐choline: DAG choline phosphotransferase; DGAT, acyl‐CoA:DAG acyltransferase; GPAT, acyl‐CoA:G3P acyltransferase; PLA, phospholipase A; LPC, phospholipase C; LPCAT, acyl‐CoA:LPC acyltransferase; PAP, PA phosphatase; PDAT, phospholipid:DAG acyltransferase.

There are two major pathways for the TAG biosynthesis in plants (Bates and Browse, 2012) (Figure 7). The *de novo* biosynthesis of TAG in plant occurs via the Kennedy pathway, in which glycerol‐2‐phosphate acyltransferase (GPAT) catalyses the conversion of glycerol‐3‐phosphate (G3P) and acyl‐CoA to lysophosphatidic acid (LPA), LPAT then converts LPA to phosphatidic acid (PA), and finally phosphatidic acid phosphatase (PAP) and DAG acyltransferase transfer PA‐derived DAG to TAG (Kennedy, 1961). Alternatively, acyl‐CoA : lyso‐phosphatidylcholine acyltransferase (LPCAT) (Stymne and Stobart, 1984; Wang *et al*., 2012) or a phospholipase A can release an acyl group from PC to generate lyso‐PC by the reverse reaction (Chen *et al*., 2011). Choline phosphotransferase (CPT), PDCT, phospholipase A (PLA) or PDAT can redirect acyl‐CoAs from PC to TAG (Hu *et al*., 2012; Lu *et al*., 2009).

LPAT transfers an acyl group from acyl‐CoA to LPA to form phosphatidic acid. It is possible that the camelina LPAT recognizes CPA poorly and may present a bottleneck for the incorporation of CPA acyl‐CoA into PA (Nlandu Mputu *et al*., 2009). In our efforts to enhance CPA accumulation in transgenic plants, we screened CPS genes from diverse sources that accumulating cyclic FAs and identified *E. coli* CPS as an effective enzyme for the production of CPA in plants. CPA accumulation was further enhanced by the co‐expression of *Sterculia foetida* LPAT to increase di‐CPA‐PC, highlighting the utility of co‐expressing an acyltransferase from mFA‐accumulating species with mFA‐synthesizing enzymes to help mitigate bottlenecks in TAG formation upon the accumulation of mFA in transgenic plants. In transgenic camelina, CPA in TAG occurs as a higher proportion of *sn‐*1/3 than *sn‐*2 compared to that of PC or DAG. This pattern was also observed for *Sterculia* seed cyclopropene fatty acid distribution (Bao *et al*., 2003). This compositional shift suggests that the metabolism of cyclopropane fatty acids in seed oils may involve several acylation steps. It is possible that expression of GPAT, DGAT, PDCT and PDATs with high specificity for PC‐CPAs, that is from organisms evolved to accumulate CPA, in EcCPS‐SfLPAT camelina lines may further enhance the flux of CPA into TAG (Burgal *et al*., 2008; van Erp *et al*., 2011; Hu *et al*., 2012; Kim *et al*., 2011; Li *et al*., 2012). In addition to stacking additional acyltransferase with preference for CPA, we may achieve further enhancements by also suppressing the endogenous homolog (i.e. PDAT1) as proved successful for ricinoleic acid accumulation (van Erp *et al*., 2011).

Lipid species are not distributed equally between different camelina seed tissues (Horn *et al*., 2013). MSI images showed that mono‐CPA‐PC primarily accumulated in the cotyledon while the di‐CPA‐PC predominantly accumulated in the embryonic axis. That the expression levels of the EcCPS and SfLPAT are not significantly different in the two tissues and that both transgenes were under the regulation of β‐phaseolin promoter suggest that other yet‐to‐be‐identified factors are responsible for this finding. For example, previous analysis of PC and TAG from wild‐type camelina seeds suggests for TAG synthesis that while DGAT1 activity likely predominates in cotyledons, PDAT activity may be higher than predicted in the embryo axis based on expression analysis and the distribution of lipid species (Horn *et al*., 2013). Further, the distribution of di‐18 : 1 PC and tri‐18 : 1 TAG in the *fad2/fae1* was heterogeneous, with more in cotyledons relative to embryonic axis. This may be related to differences in relative DGAT1/PDAT activities (DGAT1 preferring monounsaturated FAs; PDAT1 preferring di‐ or polyunsaturated FAs). This difference in preference between monounsaturated FAs and polyunsaturated FAs was also evident in camelina DGAT1‐suppressed lines, that is in which PDAT predominates in which the 18 : 3 content was substantially enhanced, especially in cotyledons where DGAT normally predominates (Marmon *et al*., 2017). In contrast, the data for CPA presented herein—that is, in the embryo axis where PDAT predominates, the levels of di‐CPA‐PC are elevated—suggest a preference for mono‐ over di‐CPA‐PC for PDAT.

Comparative transcriptome analysis was reported for developing cottonseed embryo axis and cotyledons which revealed the differential expression of a number of transcription factors, membrane‐bound lipid‐modifying enzymes and TAG assembly enzymes (Jiao *et al*., 2013). Future work will focus on identifying potential targets in camelina for expression in cotyledons to increase the accumulation of CPA.

## Conclusions

The goal of this study was to identify bottlenecks in the accumulation of modified fatty acids, specifically CPA. To achieve this, camelina seeds co‐expressing *E. coli* cyclopropane synthase and *Sterculia foetida* lysophosphatidic acid acyltransferase were generated. SfLPAT increased di‐CPA‐PC accumulation relative to mono‐CPA‐PC which resulted in increased CPA‐DAG and CPA‐TAG accumulation. Fatty acid analysis shows PC contained more than 60% CPA, compared with 26% in DAG and 12% in TAG, indicating bottlenecks in the transfer of CPA from PC to DAG, and from DAG to TAG. These bottlenecks differ from those reported for hydroxy fatty acid accumulation in Arabidopsis in which the transfer of hydroxy fatty acids from DAG to PC was identified as the limiting step (Bates *et al*., 2014).

## Experimental procedures

### Plant growth conditions and transgenic analyses

Camelina plants were grown in walk‐in growth chambers at 22°C for 16‐h photoperiod. Binary vectors containing *E. coli* CPS, GhCPS1, GhCPS2 or SfCPS only or EcCPS and SfLPAT co‐expression cassette (Yu *et al*., 2014) were introduced into *Agrobacterium tumefaciens* strain GV3101 and transferred into camelina by agrobacterium‐mediated inoculation of camelina plants at early flowering stage along via vacuum infiltration (Lu and Kang, 2008). Seeds of transformed plants were screened for DsRed fluorescence emitted upon illumination with green light from a ×5 LED flashlight (Inova) in conjunction with a 25A red camera filter as previously described (Pidkowich *et al*., 2007).

### RNA extraction and qRT‐PCR

RNA from camelina seeds of *fad2/fae1,* EcCPS and EcCPS‐SfLPAT transgenic plants was extracted according to Schultz *et al*. (1994). RNA quality and concentration were determined by gel electrophoresis and nanodrop spectroscopy. Reverse transcription (RT) was carried out using Qiagen's QuantiTect RT Kit and qPCR carried out as described in Yu *et al*. (2011). The primers for EcCPS and SfLPAT were the same as shown in our previous report (Yu *et al*., 2014). Primers for camelina *Actin* were qCsAct‐F (5′‐GTGGTTACTCTTTCACCACCACAG‐3′) and qCsAct‐R (5′‐CCAGCATTCTCAGCATACCAATCA‐3′). The gene expression levels are calculated as the percentage of its expression relative to the expression of the camelina Actin transcript.

### Fatty acid analyses

Fatty acid analyses were carried out as described (Broadwater *et al*., 2002). For total FA analysis, lipids were extracted in methanol/chloroform (2 : 1) from 100 seeds and 5 mg heptadecanoic acid (17 : 0) was added as an internal standard. Total seed lipids were converted into fatty acid methyl esters (FAMEs) in 1% sodium methoxide at 50°C for 1 h and extracted with hexane. FAMEs from single seeds were prepared by incubating the seed with 50 μL 0.2 m trimethylsulfonium hydroxide in methanol (Butte *et al*., 1982). Lipid profiles and acyl group identification were analysed on a Hewlett‐Packard 6890 gas chromatograph equipped with a 5973 mass selective detector and Agilent J&W DB 23 capillary column (30 m × 0.25 μm × 0.25 μm). The injector was held at 225°C and the oven temperature was increased from 100 to 160°C at 25°C/min, then to 240°C at 10°C/min. The FA percentage values were presented as a mean of at least three biological replicates.

### CPA distribution in the TAG, DAG and polar lipid

Total lipids were extracted from 30 mg seeds of each T4 line by homogenizing in methanol : chloroform : formic acid (20 : 10 : 1 vol/vol). The organic solvent was extracted twice with 1/2 volume of 1 m KCl and 0.2 m H_3_PO_4_. The organic phase was combined and dried under N_2_. Polar lipid, DAG and TAG were separated through a 3‐mL Supelco Supelclean LC‐Si SPE column (Sigma‐Aldrich, St. Louis, MO) with the polar lipid fraction dried under nitrogen and redissolved in chloroform. PC and PE were separated from other polar lipids by thin layer chromatography (TLC Silica gel 60 glass plate pretreated with 0.15m (NH_4_)_2_SO_4_) with acetone/toluene/H_2_O (91 : 30 : 7). Internal standard heptadecanoic acid was added to each fraction, and FAMEs were prepared with 1 mL of 1 m methanolic HCl at 90°C for 1 h and extracted with hexane. FAMEs were quantified by GC‐MS, as previously described (Yu *et al*., 2011).

### Stereospecific analysis of the fatty acid composition of PC, DAG and TAG from transgenic camelina

Stereospecific*‐*position analysis of PC was performed as described in Yu *et al*. (2014), and the stereospecific analysis of the fatty acid composition of TAG (or DAG) was performed as described by Cahoon *et al*. (2006) except that the TLC mobile phase was replaced with hexane/diethyl ether/acetic acid (70 : 30 : 1).

### MS analysis of CPA‐containing DAG

SCIEX Triple Quad™ 4500 Mass Spectrometer (Framingham, MA) was used for quantitative analysis of fatty acid distribution in CPA‐containing DAG species. CPA‐containing DAGs were detected by neutral loss of CPA (*m/z* 313.2, [M+NH_3_]) with electrospray ionization source under positive ion mode by direct infusion of DAG samples. The mass spectrometer parameters were set as follows: ionspray voltage: 5200; temperature: 150°C; declustering potential: 80; and collision energy: 40. The *m/z* values of monoacylglycerol after neutral loss were used to identify the fatty acid species other than CPA in CPA‐containing DAG. The relative amount of CPA‐containing DAG species was calculated by normalizing the monoacylglycerol peak area.

### ESI‐MS/MS analysis of TAG molecular species

ESI‐MS/MS analysis of intact, ammoniated triacylglycerols by direct infusion was performed using an Applied Biosystems QTRAP 4000 as previously described (Nguyen *et al*., 2015). Neutral loss scanning for loss of *m/z* 285.2 (c17 : 0 CPA), *m/z* 313.2 (c19 : 0 CPA), *m/z* 341.2 (c21 : 0 CPA) and *m/z* 369.2 (c23 : 0 CPA) from TAG species and product ion scans of individual TAG species were used to confirm the presence of one, two or three CPA molecules in TAGs.

### Mass spectrometry imaging

MS imaging was carried out on transgenic camelina seeds as described in Horn *et al*. (2013). Briefly, mature desiccated seeds were embedded in 10% gelatin, frozen and cryosectioned at 40‐μm sections. Tissue sections were adhered to glass microscope slides and freeze‐dried overnight. A 20 mg/mL chemical matrix of 2,5‐dihydroxybenzoic in 70 : 30 (v/v) methanol : ultrapure water was applied using the overlay method (SunChrom SunCollect MALDI spotter, Fredrichsdorf, Germany) to tissue samples. MS imaging data were acquired on a Thermo Fisher Scientific linear ion trap‐Orbitrap hybrid mass spectrometer equipped with a MALDI source (MALDI LTQ Orbitrap‐XL). Raw MS imaging data were analysed as described in Horn *et al*. (2013) using the Metabolite Imager software (Horn and Chapman, 2014).

### Accession numbers

The following genes were used in this study: EcCPS1 [NCBI Gene ID: 944811], GhCPS1 [GenBank: 574036.1], GhCPS2 [GenBank: 574037.1], SfCPS [NCBI Gene ID: 1171057]; and SfLPAT [GenBank: KC894726.1].

## Supporting information


**Figure S1** Map of binary plant expression vectors.
**Figure S2** Seed oil content as a percentage of dry weight.
**Figure S3** Germination and growth of transgenic camelina in soil (a and c) and " MS plus 1% sucrose medium (b). Seeds from control fad2/fae1 and transgenic lines collected at the same time were compared for germination testing. (c) T4 generation of camelina plants of equivalent age.
**Figure S4** EcCPS and SfLPAT expression in embryo axis and cotyledon.
**Figure S5** Neutral Loss spectra showing TAG species of elongation products of 19:0CPA including 21 : 0 CPA and 23 : 0 CPA.
**Figure S6** Desaturated CPA (19 : 1) is detected in the TAG via GC/MS, and long‐chain CPA (21 : 0) is also detected in the transgenic plants, but is a mix with 22 : 1.
