# Supplementary material for: Identification of bottlenecks in the accumulation of cyclic fatty acids in camelina seed oil
Source: Plant Biotechnol J. 2018 Jan 18;16(4):926–38. doi: 10.1111/pbi.12839 (PMC5866947; doi:10.1111/pbi.12839)
Supplement: Supplementary file 1 — Figure S1 Map of binary plant expression vectors. Figure S2 Seed oil content as a percentage of dry weight. Figure S3 Germination and growth of transgenic camelina in soil (a and c) and " MS plus 1% sucrose medium (b). Seeds from control fad2/fae1 and transgenic lines collected at the same time were compared for germination testing. (c) T4 generation of camelina plants of equivalent age. Figure S4 EcCPS and SfLPAT expression in embryo axis and cotyledon. Figure S5 Neutral Loss spectra showing TAG species of elongation products of 19:0CPA including 21 : 0 CPA and 23 : 0 CPA. Figure S6 Desaturated CPA (19 : 1) is detected in the TAG via GC/MS, and long‐chain CPA (21 : 0) is also detected in the transgenic plants, but is a mix with 22 : 1. [file PBI-16-926-s001.docx]

**Supplementary Fig. 1. Map of binary plant expression vectors.** The CPS genes from E. coli, EcCPS; Sterculia foetida, SfCPS; cotton, GhCPS1 and GhCPS2 and the LPAT from Sterculia foetidawere placed under the control of phaseolin promoter (pPhas) and phaseolin terminator (UTR). DsRED was used as the visual screenable marker. It was placed under the control of the 35S promoter and NOS terminator is indicated (DsRED); left and right borders (LB and RB) are indicated.


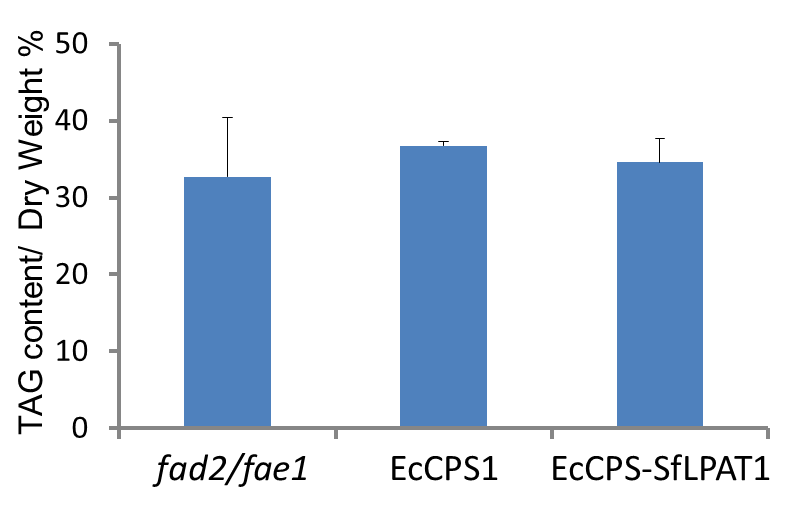


**Supplementary Fig. 2. Seed oil content as a percentage of dry weight. The values represent the mean and standard deviation of at three replicates.**

**Supplementary Fig. 3. Germination and growth of transgenic camelina in soil (a and c) and ½ MS plus 1% sucrose medium (b).** Seeds from control *fad2/fae1* and transgenic lines collected at the same time were compared for germination testing. (c) T4 generation of camelina plants of equivalent age.

**
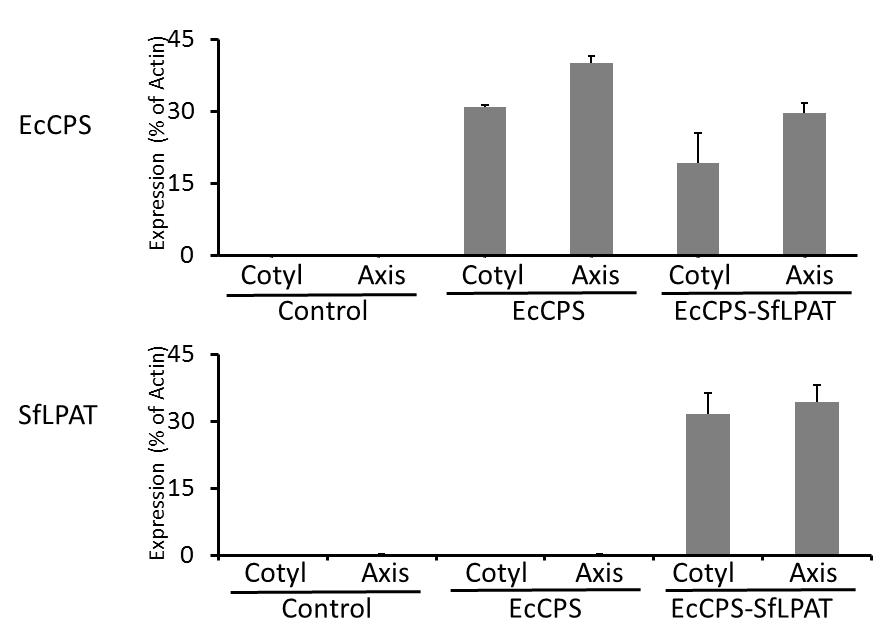
**

**Supplementary Fig. 4. EcCPS and SfLPAT expression in embryo axis and cotyledon.** qRT-PCR analysis of EcCPS and SfLPAT expression levels in axis and cotyledon of camelina *fad2/fae1* (ctr) and 3 transgenic lines harboring EcCPS or EcCPS and EcCPS-SfLPAT as indicated. The relative expression levels are reported relative to the expression of the Actin transcript. The values represent the mean and standard deviation of at least three replicates.

**Supplementary Fig. 5. Neutral Loss spectra showing TAG species of elongation products of 19:0CPA including 21:0 CPA and 23:0 CPA. (a) and (b), 21:0 CPA and 23:0 CPA. (**a)**.** NL 341.2 c21:0 confirms that 19:0CPA can be elongated to 21:0 CPA and is incorporated into TAG. *m/z* 918.8 could correspond to 16:0/18:1/21:0, *m/z* 941.0 (18:3/18:3/21:0) , *m/z* 944.9 (18:1/18:1/21:0), *m/z* 959.0 (18:1/19:0/21:0), and *m/z* 973.1 (19:0/19:0/21:0). Product ion spectra for *m/z* 944.9, *m/z* 959.0 , and 973.0 TAG confirm the presence of c21:0-containing DAGs. (c) and (d), NL 369 (c23:0 fatty acid) indicates TAG species *m/z* 973.0 and 987.0 contain 23:0 CPA. A small amount of 18:1/18:1/23:0 is present in *m/z* 973.0 TAG, and a small amount of 18:1/19:0/23:0 is present in *m/z* 986.8 TAG.

**Supplementary Fig. 6. Desaturated CPA (19:1) is detected in the TAG via GC/MS, and long chain CPA (21:0) is also detected in the transgenic plants, but is a mix with 22:1. (a)**. Red arrow point to the peak of desaturated CPA and blue arrow shows the peak of a mixture of 21:0 CPA and 22:1 in the transgenic seed. (b). Mass Spectrum of 21:0 CPA (left) and the 21:0 CPA (right).
